# Glioblastomas

**DOI:** 10.3390/cancers14010104

**Published:** 2021-12-27

**Authors:** Gaetano Finocchiaro, Giulia Berzero

**Affiliations:** 1 Neurology Unit, IRCCS San Raffaele Scientific Institute, 20132 Milan, Italy; berzero.giulia@hsr.it; 2 Vita-Salute San Raffaele University, 20132 Milan, Italy

Years ago, glioblastoma lost its second name, multiforme, which possibly was an unfortunate decision given the extraordinary heterogeneity of this overly aggressive primary brain tumor, as effectively exemplified by this Latin adjective [1]. In a way, the title of “Glioblastomas” for this Special Issue of *Cancers* attempts to recapitulate these characteristics and alludes to this deadly heterogeneity.

Eleven papers are included in this issue [2,3,4,5,6,7,8,9,10,11,12], and they range from in vitro and preclinical studies to clinical studies, and to immunotherapy and new potential treatments for glioblastomas (Figure 1). 

Arthurs and co-workers investigated the suitability of glioblastoma (GBM) cell lines as models to study GBM metabolism [2], which is preferentially based on aerobic glycolysis and lactate production, the so-called Warburg effect [13]. They found that in patient-derived cell lines, ATP-linked respiration rates and basal mitochondrial rates are lower than in normal adjacent cells from the same patients, while reserve and Krebs cycle capacities are higher. Of the five established cells lines that were tested, the T98G line was shown to better recapitulate the glycolysis-related metabolic parameters of primary GBM cells; therefore, this cell line is recommended for use in research related to glycolysis [2].

To find better representation of human GBM characteristics (such as necrosis, infiltration capacity and heterogeneity) in murine cell lines, Costa et al. generated a set of GBM cell lines via the repeated in vivo passaging of cells derived from a neural stem cell line derived from Pten/p53 double knockout mice that they previously established [14]. Cell lines were shown to be syngeneic with the immunocompetent C57/Bl6 mice: upon transplantation, they formed high-grade gliomas that recapitulated the histopathological and biological features of human GBM, including immune cell infiltration and molecular heterogeneity, as revealed by transcriptomic and genomic analysis [3]. These cell lines are available to be shared with the scientific community.

Given the significant role that epithelial to mesenchymal transition (EMT) plays in GBM resistance to treatments, Guelfi et al. investigated the relevance of two transcription factors involved in EMT, SLUG and TAL1, in GBM stem-like cells (GSC) [4]. SLUG expression was upregulated upon Notch1 activation and TGF-beta 1 treatment. A truncated isoform of TAL1, TAL1-PP22, was also upregulated upon Notch1 activation. Notably, SLUG1 and truncated TAL1 were overexpressed in GBM specimens in mutually exclusive subpopulations of cells, i.e., perivascular and endothelial cells, respectively [4]. Further study is required to uncover the translational relevance of these findings for use in therapeutic targeting.

Another report investigated GSC and corresponding tumors [5]. The authors found that in mesenchymal tumors and the derived GSCs, interferon (IFN) I and II signaling levels were high, coupled with STAT1 signaling and associated with poor survival. Chronic inhibition of the pathway in these GSCs inhibited cell proliferation and mesenchymal signatures. IFN-beta exposure induced apoptosis in GSCs with high IFN-STAT1 signaling levels; this effect was inhibited by ruxolitinib, a pan-JAK-STAT pathway blocker, or by STAT1 knockdown. Thus, this study supports the role of IFN-beta as a potential treatment targeting this GBM subtype.

A different signaling pathway in U87 and T98G GBM cells was analyzed by another paper in this series, and was shown to be based on interactions of angiotensin II and angiotensin II type I receptor (AGTR1) [6]. AngII/AGTRI signaling enhanced estrogen production through the upregulation of two aromatase gene promoters and transactivated estrogen receptor-alpha through mitogen-activated protein kinase activation. The potential clinical relevance of these observations was supported by the inverse correlation found between aromatase expression and GBM survival. AngII may contribute to the immune suppression of the tumor microenvironment by stimulating the expression of the Programmed Death-Ligand 1 (PD-L1), as also shown in non-small cell lung carcinoma [15]. The Ang II inhibitor Losartan reversed PD-L1 expression. This, coupled with decreased expression of pro-tumorigenic factors such as VEGF, bFGF and PDGF via losartan, previously demonstrated in C6 rat gliomas [16], may support the idea of repurposing this drug for use in anti-glioma treatment.

On a clinical level, Jabbarli et al. analyzed correlations between different anti-epileptic drugs (AEDs) and patient survival rates in 872 GBM patients treated at the University Hospital of Essen in Germany, from 2006 to 2018 [7]. A meta-analysis of seven other studies was also performed, meaning a total of 5614 patients were considered. In the institutional cohort, concomitant radio-chemotherapy followed by adjuvant chemotherapy, which is the present standard treatment for GBM [17], was initiated in 74% of the patients. A total of 295 (33.8%) cases were treated with AEDs. The data showed that only levetiracetam treatment was associated with favorable overall survival (OS) rates when compared with other AED treatments (*p* = 0.004) or no levetiracetam (*p* < 0.0001). The results of the meta-analysis confirmed improved survival in the presence of levetiracetam (*p* = 0.02). In both scenarios, levetiracetam was associated with longer OS rates, also at multivariate Cox regression analysis. Previous data showed that levetiracetam may enhance p-53-mediated MGMT inhibition, thus sensitizing GBM cells to temozolomide [18]. This may provide an intriguing background to the observations of Jabbarli et al; however, we should remember that a large-scale analysis of 1869 patients from four randomized studies, including temozolomide treatment, failed to demonstrate an advantage with regard to survival in patients treated using levetiracetam vs. other AEDs or no AEDs [19].

Another clinical paper in this Special Issue reviews palliative care (PC) service utilization and advanced care planning (ACP) in GBM patients [8]. Sixteen articles were selected, all from non-randomized studies conducted in six countries, mostly published in 2014 or later. ACP documentation varied from 4–55%, PC referral was pursued in 39–40% of cases, and hospice referrals were made for 66–76% of patients. Hospitalizations frequently occurred at the end of life, with 20–56% of patients spending over 25% of their overall survival time hospitalized. Many GBM patients did not pursue ACP or did not have access to PC. The review included data from US, Canadian and European studies that could also be evaluated considering previous observations obtained by comparisons of different patterns of palliative care in Asia, Europe, and North America [20]. 

The final four papers in this Special Issue deal with immunotherapy or potentially novel treatments for glioblastomas. Jin et al. built on data supporting the adenosinergic pathway (AP) as a major suppressor of antitumor immune responses in the GBM microenvironment [9]. Tumor cells evolve to metabolize pro-inflammatory ATP to anti-inflammatory adenosine that can suppress immune responses through the signaling of adenosine receptors on immune cells. Preclinical results targeting AP in GBM showed promising results in reinvigorating antitumor responses, overriding chemoresistance, and increasing survival. The authors suggest that future clinical studies should consider AP in combination therapies with other immunotherapeutic approaches.

Andersen et al. reviewed the role of tumor-associated macrophages (TAMs) in the GBM microenvironment [10]. While TAMs have been classified as M1 and M2 phenotypes, recent studies based on single-cell technologies have identified expression pattern differences; the results of these studies are beginning to provide us with a deeper understanding of the heterogeneous subpopulations of TAMs in glioblastomas.

Aguilar et al. focused on the application of electric fields for the treatment of cancers [11]. They outlined the clinical impact of tumor treating fields (TTFields) on the treatment of cancers such as GBM. The “standard” mechanism of action of TTFields is based on their potential to disrupt the formation and segregation of the mitotic spindle in actively dividing cells. Besides this, however, TTFields may alter the functionality and permeability of cancer cell membranes. Three mechanistic models may explain the more recent observations regarding alternating electric fields (AEFs) effects: the voltage-gated ion channel, bio-electro-rheological, and electroporation models [11].

Finally, Ranjan et al. discussed targeting cyclin-dependent kinase 9 (CDK9), one of the CDKs that regulate transcription by RNA Polymerase II (RNA Pol II), as an emerging therapeutic approach that has the potential to overcome challenges in GBM management [12]. Specifically, they discussed how CDK9 inhibition can impact transcription, metabolism, DNA damage repair, epigenetics, and the immune response and therefore facilitate anti-GBM responses. The potential of small-molecule inhibitors of CDK9 in clinical trials for GBM patients was also discussed.

More than 15 years on from the definition of the standard treatment of GBM by Stupp et al. [21], we are still struggling to identify a novel line of treatment that benefits a higher number GBM patients. The papers included in this Special Issue provide a good oversight into the complexity of such endeavor but also give hints toward pathways that can be followed to get closer to the achievement of this goal. 

## Figures and Tables

**Figure 1:**
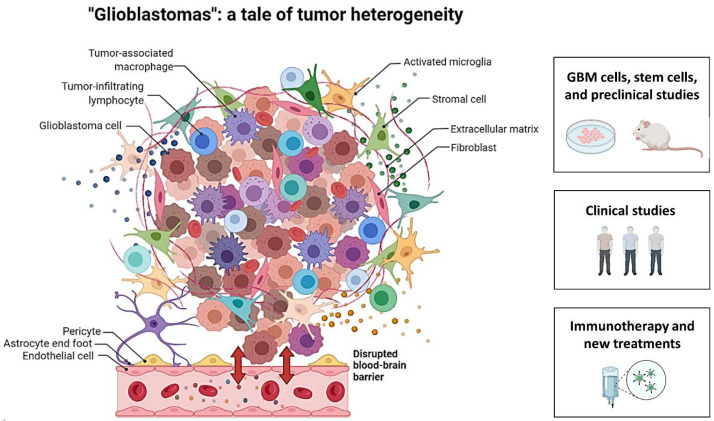
On the left, an illustration of the different cell populations composing glioblastoma and its microenvironment, as well as associated blood-brain barrier disruption. On the right, a summary of the different areas covered by the articles in this Special Issue: GBM cells, stem cells, and preclinical studies [2,3,4,5,6], clinical studies [7,8], immunotherapy and new treatments [9,10,11,12]. Created with BioRender.com.
